# Clinical Insights into Sickle Cell Disease: A Comprehensive Multicenter Retrospective Analysis of Clinical Characteristics and Outcomes Across Different Age Groups

**DOI:** 10.3390/jcm13237224

**Published:** 2024-11-28

**Authors:** Daniyah A. Almarghalani, Renad A. Alotaibi, Teef T. Alzlami, Ozouf F. Alhumaidi, Najla M. Alharthi, Fatimah M. Alboqami, Khulood A. Almehmadi, Samar F. Miski, Ali Alshahrani, Faisal F. Alamri, Khadeejah Alsolami, Suhaib Mamduh Doman, Maha T. Alhamdi, Areej Zubaid, Wasan S. Aloufi

**Affiliations:** 1 Department of Pharmacology and Toxicology, College of Pharmacy, Taif University, P.O. Box 11099, Taif 21944, Saudi Arabia; renad5xi@gmail.com (R.A.A.); taifalzlami@gmail.com (T.T.A.); ozouf.alhumaidi@gmail.com (O.F.A.); jool-66@hotmail.com (N.M.A.); alboqamii84@gmail.com (F.M.A.); k.alsolami@tu.edu.sa (K.A.); maha_talal@hotmail.com (M.T.A.); areej.zubaid@gmail.com (A.Z.); 2 Stroke Research Unit, Taif University, P.O. Box 11099, Taif 21944, Saudi Arabia; 3 King Abdulaziz Hospital, Taif 26521, Saudi Arabia; 4 Al Hada Military Hospital, Taif 26792, Saudi Arabia; 5 Department of Pharmacology and Toxicology, Faculty of Pharmacy, King Abdulaziz University, Jeddah 21589, Saudi Arabia; kaalmehmadi@kau.edu.sa; 6 Department of Pharmacology and Toxicology, College of Pharmacy, Taibah University, Medina 41411, Saudi Arabia; smiski@taibahu.edu.sa; 7 Department of Clinical Pharmacy, College of Pharmacy, Taif University, P.O. Box 888, Haweiah 21974, Saudi Arabia; a.shahrani@tu.edu.sa; 8 Department of Basic Sciences, College of Science and Health Professions, King Saud bin Abdulaziz University for Health Sciences, Jeddah 21582, Saudi Arabia; alamrif@ksau-hs.edu.sa; 9 King Abdullah International Medical Research Center, Jeddah 22384, Saudi Arabia; 10 Al Wajh General Hospital, Al Wajh 48721, Saudi Arabia; smhhd@hotmail.com; 11 Taif Children’s Hospital, Taif 26514, Saudi Arabia; wasansaad2@gmail.com

**Keywords:** sickle cell disease (SCD), different age groups, clinical characteristics, complications, treatment

## Abstract

**Background:** Sickle cell disease (SCD) is a genetic hematological disorder associated with significant mortality and a range of complex complications that manifest differently across various age groups. **Methods:** This study aimed to evaluate the demographic, clinical, and laboratory characteristics of SCD patients in Taif City, Saudi Arabia, with a focus on variations among children, adolescents, adults, and middle-aged individuals. A multicenter retrospective cohort study included 129 patients with confirmed diagnosis of SCD between January 2018 to October 2023 and divided into 4 cohorts. The analysis compared hospital stay durations, admission rates, SCD complications, and medication usage. **Results:** Among the participants, 35 were children (27%), 18 adolescents (14%), 63 adults (49%), and 13 middle-aged individuals (10%). Clinical complications as splenic disease in children (34.3%) were more frequent compared to adolescents (5.6%) and adults (4.8%). Additionally, chronic kidney disease was more prevalent in middle-aged patients (15.4%). Pain was reported in 65.1% of patients, with vascular occlusive crises occurring in 41.1%. Treatment adherence varied, with children showing higher penicillin use (74.3%), while opioid usage was greater in middle-aged patients (76.9%). **Conclusions:** The findings underscore the necessity for age-specific management strategies in SCD. Further research with larger populations is suggested to enhance the understanding of disease progression and treatment efficacy across different age groups.

## 1. Introduction

Sickle cell disease (SCD) is a genetic hematological disorder resulting from a mutation in the beta-globin gene, which leads to the production of abnormal hemoglobin S (HbS) [1,2]. The prevalence of SCD varies globally; in the United States, the approximate prevalence is 329 cases per 1,000,000 people [3]. In the United Kingdom, it is estimated to be around 217 cases per 1,000,000 [4]. The prevalence is significantly higher in populations of African and Middle Eastern origins, notably in Nigeria, where SCD affects over 20,000 per 1,000,000 [5]. The prevalence is even more pronounced in Saudi Arabia, with an estimated incidence of about 4.51%, or 45,100 cases per 1,000,000 among adults, and 0.24%, or 2400 per 1,000,000 among children and adolescents. SCD is particularly dominant in the eastern and southwestern regions of Saudi Arabia [6,7].

Clinically, SCD manifests with a spectrum of complications, including vaso-occlusive crises, chronic hemolytic anemia, and other complications that significantly affect the quality of life and longevity of affected patients [8,9,10]. A vaso-occlusive crisis occurs when sickle-shaped red blood cells (RBCs) obstruct small blood vessels, causing severe pain and damage to the affected tissues. These episodes can happen unpredictably and result in organ ischemia, including damage to the lungs, kidneys, liver, and spleen [11]. Chronic anemia is another common complication, as abnormal RBCs have a shorter lifespan and are prone to hemolysis, leading to fatigue, shortness of breath, and decreased oxygen-carrying capacity [12]. In addition, SCD increases the risk of infections, particularly with encapsulated bacteria, due to functional asplenia and impaired immune function [13]. Patients with SCD may experience complications related to impaired blood flow such as stroke (ischemic stroke and hemorrhagic stroke), delayed growth and development, cardiovascular diseases, mental disorders, pulmonary hypertension, acute chest syndrome, eye complications, gallbladder disease, and leg ulcers. These complications can notably impact the quality of life and overall health of patients with SCD, requiring comprehensive medical management and supportive care [14,15,16,17].

SCD clinical severity and life expectancy have increased in recent years in higher-resource countries such as the USA. In 1994, the median death age for women and men was 48 and 42 years of age, respectively. In 2019, the median death age was reported to be 54 years [18]. Unfortunately, patients in low-resource settings continue to face high mortality rates [19,20]. This demographic shift necessitates a comprehensive understanding of how SCD affects patients across their lifespan, from childhood through adulthood. The integration of clinical data from multiple centers allows for a more robust analysis of the disease’s impact, reflecting the diverse experiences of patients in various age groups.

Moreover, the clinical presentation of SCD does vary across different age groups and is influenced by factors such as genetic background, environmental conditions, and access to healthcare [21,22,23]. For instance, pediatric patients often exhibit distinct complications, including increased susceptibility to infections, acute chest syndrome, and splenic sequestration [24,25]; however, adults may face chronic issues such as organ damage and chronic organ complications [2,17,26]. Understanding these age-related differences is crucial for optimizing treatment and improving patient outcomes.

Recent and current research studies have focused on diagnosing SCD early and treating it holistically in different age categories [8]. Multidisciplinary approaches to managing SCD are essential for optimizing patient outcomes and enhancing the quality of life across various age groups [27]. Pharmacological treatments, such as hydroxyurea, blood transfusions, and pain management strategies, play a crucial role in this comprehensive care model [28,29,30]. Nevertheless, these treatments attain varying levels of effectiveness within different age populations. For example, children may require hydroxyurea to boost fetal hemoglobin to decrease the incidence of vaso-occlusive crises. [31]. Azmet et al. demonstrated that children under 14 years old who were treated with hydroxyurea experienced a significant reduction in episodes of vaso-occlusive crisis and length of stay in the hospital [32]. In addition, this treatment led to fewer hospitalizations and notable improvements in complete blood count indices [32]. In contrast, adults potentially require more intensive management of chronic complications such as organ damage, often necessitating different pharmacological interventions [33]. Furthermore, adherence to treatment varies across different ages, with children typically showing better adherence when supported by caregivers. Adults may face barriers to access and self-management [34,35,36].

In the current study, we hypothesize that there are significant variations in demographic, clinical, and laboratory characteristics, as well as complications and management of SCD patients, across different age groups over a five-year period. Furthermore, treatment patterns and clinical outcomes, such as hospitalizations and pharmacological and non-pharmacological treatment, are expected to be significantly different among these age groups. Therefore, the study aims to provide valuable insights and a comprehensive analysis of patient features and outcomes across different age groups.

## 2. Methods

### 2.1. Study Design and Setting

A multicenter retrospective cohort study was conducted at Alhada Military Hospital, King Faisal Hospital, King Abdulaziz Hospital, and Children’s Hospital in Taif, Saudi Arabia, between January 2018 and October 2023. This multicenter approach facilitated the collection of a comprehensive dataset that accurately reflected the demographics of the western region of Saudi Arabia. Each facility offers clinical care to Saudi and non-Saudi residents, ensuring diverse representation within the patient population. Ethical approval was obtained from the Taif Health Ministry (IRB Registration Number: HAP-02-T-067). Patients were enrolled without consent due to the retrospective nature of the study.

### 2.2. Study Participants

**Figure 1:**
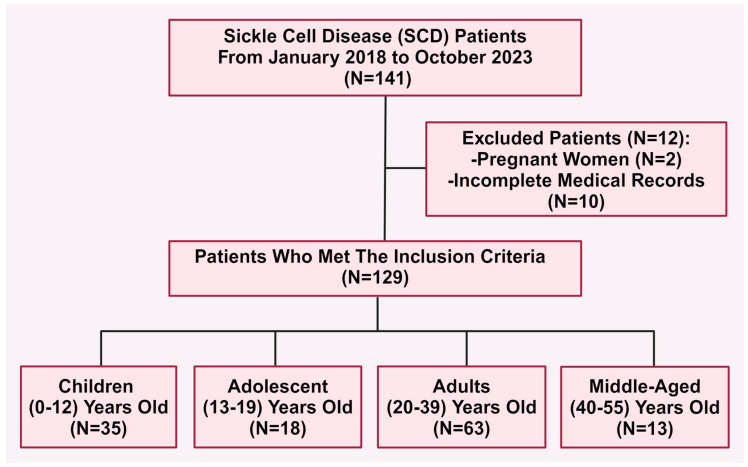
The flowchart illustrates the study population and the criteria for selecting eligible patients with sickle cell disease (SCD) across various age groups, including children, adolescents, adults, and middle-aged individuals.

Paper and electronic medical records using unique medical record numbers for each patient were used to identify patients’ eligibility based on established inclusion and exclusion criteria. Inclusion criteria encompassed all patients diagnosed with SCD of any age and gender, including those with known hemoglobinopathies involving one or two genes related to sickle hemoglobin. Patients excluded are pregnant women and those with incomplete records (Figure 1).

### 2.3. Outcomes

The primary outcomes of this study include the baseline characteristics of patients with SCD categorized by different age groups along with their laboratory and clinical characteristics, focusing on the length of hospital stays and the number of hospital and ICU admissions. Additionally, the study aims to identify and characterize the complications associated with SCD across these age groups. Secondary outcomes are the management approaches employed for SCD patients, examining how treatment strategies vary among different age groups.

### 2.4. Data Collection

The data collected for this study included demographic information, length of hospital stay, number of hospital admissions, number of intensive care unit (ICU) admissions, oxygen (O_2_) saturation levels, and laboratory results (e.g., white blood cell count (WBC), reticulocyte count, red blood cell count (RBC), platelets, hematocrit, hemoglobin, creatinine, alanine aminotransferase (ALT), aspartate aminotransferase (AST), total bilirubin, direct bilirubin, and lactate dehydrogenase (LDH)). In addition, we also collected data on complications that are typically associated with SCD, including acute chest syndrome, avascular osteonecrosis, acute kidney disease (AKD), chronic kidney disease (CKD), pulmonary hypertension, diabetes, hypertension, delayed growth and development, immunosuppression, pain, ischemic stroke, osteomyelitis, shortness of breath, osteoporosis, gallstones, epilepsy, urinary tract infections (UTIs), jaundice, acute cholecystitis, hemolytic crisis, priapism, splenic disease, vascular occlusive crises, and mortality. Lastly, information on both pharmacological and non-pharmacological treatments was also collected.

### 2.5. Statistical Analysis

A descriptive analysis was performed to summarize the demographic and clinical characteristics of the study participants across different age groups. Frequencies and percentages were calculated for categorical variables, while means with standard deviations were computed for numeric variables. Comparisons of categorical variables across age groups were assessed using the chi-square test or Fisher’s exact test. Numeric variables were evaluated using either the independent *t*-test or one-way ANOVA, as appropriate. Data analysis was conducted using the Statistical Package for the Social Sciences (SPSS) software (version 30.0.0).

## 3. Results

### 3.1. Patient Demographics

**Figure 2:**
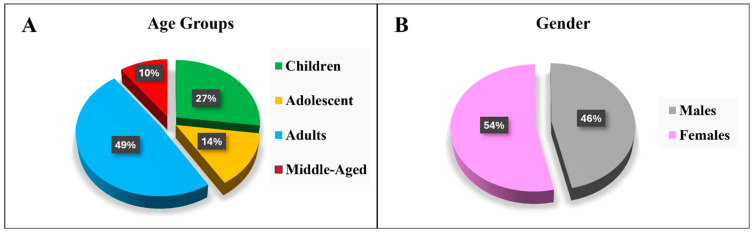
Description of (**A**) age groups and (**B**) gender in all studied patients.

Between January 2018 and October 2023, data were collected from 129 patients who met the inclusion criteria. Of these, there were 35 children (27%), 18 adolescents (14%), 63 adults (49%), and 13 in the middle-aged category (10%) (Table 1 and Figure 2A). The average age of all the patients in the study was 23 years, with a standard deviation of 13 (mean ± SD = 23 ± 13) (Table 1). Among the population, 60 were male (46.5%) and 69 were female (53.3%) (Table 1 and Figure 2B) with no statistically significant differences observed among all age groups (*p* = 0.7) (Table 2).

### 3.2. Clinical Characteristics

Clinical characteristics were varied by age group. The average length of hospital stay was significantly different among the groups, with children staying an average of 3.7 days, adolescents 9.6 days, adults 5.4 days, and middle-aged patients 3.9 days (F = 2.982, *p* = 0.034). The number of ICU admissions differed significantly, with adults and middle-aged patients having lower rates (0.1 and 0.0, respectively) compared with children (1.0) and adolescents (0.2) (F = 4.727, *p* = 0.004). Oxygen saturation levels showed no significant differences across age groups (F = 0.31, *p* = 0.82) (Table 3).

### 3.3. Laboratory Characteristics

Laboratory findings were also altered by age. The mean white blood cell count was highest in adults (14.6) and middle-aged patients (14.9) but not statistically significant (F = 1.76, *p* = 0.16). Creatinine levels were significantly higher in middle-aged patients (2.2) (F = 4.416, *p* = 0.006), while AST levels were significantly elevated in adolescents (78.6) (F = 5.606, *p* = 0.001). LDH levels also differed significantly, with adolescents having the highest mean at 1226 (F = 4.950, *p* = 0.004). However, the other reported laboratory parameters showed no statistically significant differences (*p* > 0.05) among all studied groups (Table 4).

### 3.4. Complications Associated with SCD

The most frequent complications observed in all studied patients were pain (84 patients, 65.1%), vascular occlusive crisis (53 patients, 41.1%), acute chest syndrome (16 patients, 12.4%), splenic diseases (16 patients, 12.4%), gallstones (10 patients, 7.8%), and jaundice (7 patients, 5.4%) (Supplementary Table S1 and Figure S1).

A comparison of complications across age groups showed that pain was prevalent in 68.6% of children, 55.6% of adolescents, 66.7% of adults, and 61.5% of middle-aged patients. Significant differences were found in the prevalence of splenic disease, observed in 34.3% of children but only 5.6% of adolescents and 4.8% of adults (X^2^ = 21.4, *p* < 0.001). Chronic kidney disease was more common in middle-aged patients (15.4%) compared with adults (1.6%) (X^2^ = 11.2, *p* = 0.01). Other complications showed no significant differences across age groups (Table 5).

### 3.5. Medical Co-Morbidities and Mortality Reported with SCD

The co-morbidities experienced by patients included diabetes and hypertension. Among the 35 children, diabetes was reported in 1 (2.9%), while none of the 18 adolescents had the condition. In the adult group (*n* = 63), 3 (4.8%) were reported with diabetes, and 1 (7.7%) of the 13 middle-aged patients was affected. The comparison showed no significant differences in diabetes prevalence across age groups (X^2^ = 1.5, *p* = 0.7). Hypertension was absent in children and adolescents but reported in 3 (4.8%) of the adults and 2 (15.4%) of the middle-aged patients, indicating a trend without significant difference (X^2^ = 6.9, *p* = 0.08). Mortality was reported in 1 (2.9%) child and 1 (5.6%) adolescent, with no deaths among the adult or middle-aged patients, reflecting no significant differences in mortality rates (X^2^ = 3.5, *p* = 0.32) (Supplementary Table S2).

### 3.6. Pharmacological Treatment Pattern Related to SCD

The comparison of pharmacological treatments among different age groups revealed notable findings. Folic acid was widely used, with 88.6% of children, 88.9% of adolescents, 77.8% of adults, and 84.6% of middle-aged individuals reported using it, though differences were not statistically significant (X^2^ = 2.45, *p* = 0.49). Hydroxyurea usage varied slightly among groups, used in 62.9% of children, 61.1% of adolescents, 50.8% of adults, and 53.8% of middle-aged patients, also showing no significant differences (X^2^ = 1.57, *p* = 0.67). Angiotensin-converting enzyme (ACE) inhibitors were notably used by 15.4% of middle-aged and 1.6% of adult patients, with no usage reported in the other age groups, indicating a significant difference (X^2^ = 11.2, *p* = 0.011). Erythropoietin (EPO) was rarely utilized, with only one child (2.9%) using it and no reports in other groups (X^2^ = 2.7, *p* = 0.44). Penicillin use was significantly higher in children (74.3%) than adolescents (38.9%), adults (19.0%), and only 7.7% middle-aged patients (X^2^ = 34.9, *p* < 0.001). Iron chelation therapy was reported in 11.4% of children, with no instances in other age groups, indicating a significant difference (X^2^ = 11.1, *p* = 0.011). Lastly, the usage of opioids was significantly higher among adults (65.1%) and middle-aged patients (76.9%) compared with children (20.0%) and adolescents (61.1%) (X^2^ = 22.5, *p* < 0.001) (Table 6).

### 3.7. Non-Pharmacological Treatment Patterns Related to SCD

The analysis of non-pharmacological treatment across age groups demonstrated several key findings. Oxygen therapy was used infrequently: 14.3% of children, 11.1% of adolescents, and 6.3% of adults. No middle-aged patients were reported using it (X^2^ = 3.2, *p* = 0.36). Additionally, only one patient from the adult group received hematopoietic cell transplants (X^2^ = 1.06, *p* = 0.79). In contrast, blood transfusion was more common, occurring in 71.4% of children, 61.1% of adolescents, 44.4% of adults, and 76.9% of middle-aged patients. This indicates significant differences across the age groups (X^2^ = 11.02, *p* = 0.012) (Table 7).

### 3.8. Vaccine Analysis in Patients with SCD

Results and Supplementary Table S3 are included in the Supplementary Data.

## 4. Discussion

This study presents an in-depth analysis of the clinical features, complications, and treatment approaches associated with sickle cell disease (SCD) across different age groups in Taif City, Saudi Arabia. The findings reveal significant insights into how SCD manifests differently throughout the lifespan, highlighting the need for effectively categorizing management strategies to address these variations.

The distribution of patients across age groups shows a considerable adult population, with 49% adult participants, while only 27% children, 14% adolescents, and 10% middle-aged. This aligns with findings from other studies that indicate a similar demographic trend, where adults represent a substantial portion of the SCD population, often due to advances in medical care that extend life expectancy [37,38]. The average age of patients in this study was 23 years, suggesting that many patients with SCD are surviving into adulthood, emphasizing the need for ongoing care strategies that address the specific challenges faced by middle-aged and old patients. In addition, the gender distribution in this cohort showed a slight predominance of female (53.3%) over male (46.5%) patients, although the difference was not statistically significant (*p* = 0.7). This finding is consistent with some of the literature that reports a balanced gender ratio among SCD patients [37,39,40]. However, variations in gender ratios may arise due to geographic and socioeconomic factors as well as the complexities of complications that can affect access to healthcare and the management of the disease [41,42,43]. Understanding these dynamics is crucial for developing targeted interventions considering gender-specific health needs.

The average length of hospital stay varied notably among the age groups, with children averaging 3.7 days, adolescents 9.6 days, adults 5.4 days, and middle-aged patients 3.9 days (F = 2.982, *p* = 0.034). The more extended hospital stays observed in adolescents may reflect the complexity of their clinical presentations, including the occurrence of severe pain crises and complications. A study aligned with our findings indicated that for the age group of 8 to 12 years (*n* = 22), the mean length of hospital stay was shorter, at 3.23 days, compared with 5.85 days for the 13- to 19-year-old group (*n* = 60), suggesting a significant correlation between a higher initial number of body sites experiencing pain and an extended length of hospital stay (r = 0.39; *p* < 0.001) in the 13- to 19-year-old group [44]. Adolescents are also in a transitional phase, where they may struggle with adherence to treatment regimens, potentially leading to more severe exacerbations of SCD [45,46]. Conversely, the shorter hospital stays for children might indicate a more acute presentation of the disease with potentially fewer chronic complications at this stage [44]. Moreover, the significant differences in ICU admissions further illustrate the varying severity of SCD across age groups. Children experienced the highest rate of ICU admissions (1.0), compared with adolescents (0.2) and adults (0.1), with middle-aged patients showing no admissions (0.0) (F = 4.727, *p* = 0.004). This trend may reflect the acute nature of SCD complications in younger patients, such as severe vaso-occlusive crises and bacterial or viral infections, which often necessitate intensive care intervention [13]. For instance, in sub-Saharan Africa, approximately 50% of children with sickle cell disease (SCD) succumb to infections before reaching the age of five. Additionally, these children are over 50 times more likely to experience invasive pneumococcal disease compared with their peers [5,47].

In contrast, the lower ICU admission rates in adults and middle-aged patients may suggest a progression toward chronic management strategies that allow for less acute intervention [48]; however, it also raises questions about potential undertreatment or inadequate emergency care in these populations [8]. Interestingly, oxygen saturation levels showed no significant differences across the age groups (F = 0.31, *p* = 0.82). This finding indicates that, although patients with SCD may present with different clinical symptoms and hospitalization profiles, respiratory impairment is an important issue in this population and does not significantly change with age. This could indicate that the acute management of pain and other complications effectively goes a long way in preserving oxygenation, regardless of age. However, it also emphasizes the need for ongoing respiratory monitoring in all SCD patients, as hypoxia can occur during crises or acute chest syndrome and may not be immediately apparent [49,50].

The laboratory findings in this study reveal significant age-related differences in various hematological and biochemical parameters among patients with SCD. These variations notably emphasize the importance of considering age in assessing disease severity and treatment approaches. The mean white blood cell count was highest in adults (14.6) and middle-aged patients (14.9), although this difference was not statistically significant (F = 1.76, *p* = 0.16). Elevated white blood cell counts in SCD patients potentially can and often do indicate an inflammatory response or infection, which are common complications of the disease [51]. While the lack of statistical significance suggests that age may not be a primary factor influencing white blood cell counts, it is essential to note that monitoring these levels closely has the potential to provide valuable insights into a patient’s clinical status and potential complications. Moreover, creatinine levels were significantly elevated in middle-aged patients (2.2) (F = 4.416, *p* = 0.006), which, in turn, may suggest renal impairment. This concurs with earlier studies indicating that CKD was prevalent in SCD because of recurrent vaso-occlusive crises and hemolysis, which cause progressive renal damage [52,53]. Regular monitoring of renal function in this group is crucial for early intervention and management of CKD. Adolescents also exhibited significantly elevated AST levels (78.6) (F = 5.606, *p* = 0.001), which could indicate liver involvement or hemolysis-related liver dysfunction. Elevated AST in this group may reflect the increased incidence of hemolysis and other complications frequently occurring during adolescence [54,55]. Furthermore, LDH levels were notably higher in adolescents, with a mean of 1226 (F = 4.950, *p* = 0.004). Elevated LDH is commonly associated with hemolysis and tissue injury, suggesting that adolescents may experience more frequent or severe vaso-occlusive episodes that contribute to this elevation [56,57].

This study provides important insights into the complications of SCD within the different patient age populations. Pain was identified as the most common complication, affecting 65.1% of patients, followed by vascular occlusive crises (41.1%), acute chest syndrome (12.4%), splenic diseases (12.4%), gallstones (7.8%), and jaundice (5.4%). These findings are consistent with existing studies emphasizing pain and vaso-occlusive crises as major challenges for SCD patients [58,59]. The high prevalence of pain highlights its significant impact on quality of life across all age groups, with variations that may reflect differences in disease progression and pain management practices. Notably, a substantial prevalence of splenic disease was found in children (34.3%) compared with lower rates in adolescents (5.6%) and adults (4.8%), indicating that children are particularly vulnerable to splenic complications, which tend to decrease with age. This finding highlights the vulnerability of children with SCD to splenic complications, which can be attributed to recurrent splenic infarctions, leading to functional asplenia [60,61]. Furthermore, chronic kidney disease (CKD) was more prevalent in middle-aged patients (15.4%) compared with adults (1.6%), underscoring the need for regular screening and proactive management to address renal complications in older SCD patients. This finding does, in a notable way, correlate with a significant increase in creatinine levels in the middle-aged group. It is important to note that it is particularly concerning, considering that CKD is a common complication of SCD that most of the time results from recurrent vaso-occlusive events and hemolysis, which contribute to renal damage over time [53]. In a retrospective study involving 98 patients over a five-year period, Gosmanova et al. demonstrated that higher albuminuria and creatinine levels are significantly associated with the progression of chronic kidney disease (CKD) [62]. During this time, the prevalence of CKD rose from 29% to 42%, with significant increases observed in the number of patients across stages 1, 2, 4, and 5 of the disease [62]. These findings indicate that the prevalence of CKD increases with age [62].

The findings regarding co-morbidities in patients with SCD reveal essential information regarding the health challenges of different age groups. In this study, diabetes was reported in only 2.9% of children, 4.8% of adults, and 7.7 of middle-aged patients, with no cases in adolescents. The low prevalence of diabetes across the age groups suggests that metabolic complications may not be as prominent in younger patients with SCD, aligning with previous research indicating that chronic complications tend to emerge later in life [63]. Hypertension was notably absent in children and adolescents but was present in 4.8% of adults and 15.4% of middle-aged patients, indicating a potential trend toward increasing cardiovascular complications with age; however, this was not statistically significant (X^2^ = 6.9, *p* = 0.08). This observation aligns with other studies that have highlighted the increased risk of hypertension in older adults with SCD, likely due to cumulative organ damage and increased vascular resistance associated with chronic hemolysis and inflammation [26,64,65,66]. Moreover, the mortality rates in this study reflected a low incidence, with 2.9% of children and 5.6% of adolescents reported as deceased, while no deaths occurred among adults or middle-aged patients (X^2^ = 3.5, *p* = 0.32). This finding is consistent with the existing literature that indicates improved survival rates among SCD patients due to advancements in management and care [67,68].

The analysis of pharmacological treatments across different age groups in this study reveals essential patterns in the management of SCD. Folic acid emerged as a widely utilized supplement, with high usage rates across all age groups: 88.6% in children, 88.9% in adolescents, 77.8% in adults, and 84.6% in middle-aged groups. However, the differences in usage were not statistically significant (X^2^ = 2.45, *p* = 0.49), suggesting a consistent recognition of the importance of folic acid in supporting erythropoiesis across all age demographics [62]. Moreover, hydroxyurea, a key therapeutic agent for reducing SCD, demonstrated varied usage rates: 62.9% in children, 61.1% in adolescents, 50.8% in adults, and 53.8% in middle-aged patients. Again, these differences were not statistically significant (X^2^ = 1.57, *p* = 0.67), indicating a general acceptance of hydroxyurea across age groups, although the slightly lower rates in adults and middle-aged patients may reflect hesitancy or specific clinical considerations in these age groups [69,70]. A notable finding was the use of ACE inhibitors, which were reported in 15.4% of middle-aged patients and only 1.6% of adults, with no usage in the younger cohorts. This significant difference (X^2^ = 11.2, *p* = 0.011) suggests a targeted approach to managing hypertension or renal complications in older patients, as they may be more prevalent in this demographic [65,71]. Erythropoietin (EPO) was rarely utilized, with only one child (2.9%) using it and no reports in the older age groups (X^2^ = 2.7, *p* = 0.44). This underutilization may reflect the current guidelines and practices surrounding EPO, which is generally recommended in specific cases of anemia rather than as a standard treatment for SCD [72,73]. Penicillin prophylaxis showed significant variations, being used by 74.3% of children, 38.9% of adolescents, 19.0% of adults, and only 7.7% of middle-aged patients (X^2^ = 34.9, *p* < 0.001). This trend highlights the importance of penicillin in preventing infections in younger patients, especially in the context of functional asplenia common in SCD, and reflects a decline in its use as patients age, possibly due to the perceived lower risk of pneumococcal infections in older patients [24,74,75]. Iron chelation therapy was reported in 11.4% of children but not in any other age group (X^2^ = 11.1, *p* = 0.011). This finding suggests that iron overload may be a concern primarily in younger patients, potentially due to repeated blood transfusions, necessitating careful monitoring and management [76]. Lastly, the significant differences in opioid usage were striking, with higher rates noted among adults (65.1%) and middle-aged patients (76.9%) compared with children (20.0%) and adolescents (61.1%) (X^2^ = 22.5, *p* < 0.001), suggesting the need for effective pain management strategies in older patients, who may experience more severe and chronic pain due to SCD, while also indicating a cautious approach to opioid use in younger populations [77,78,79,80].

Analyzing non-pharmacological treatments for SCD across different age groups showed several noteworthy trends. Oxygen therapy was minimally utilized; only 14.3% of children, 11.1% of adolescents, 6.3% of adults, and no middle-aged patients utilized oxygen therapy (X^2^ = 3.2, *p* = 0.36), highlighting that complications such as acute chest syndrome or severe hypoxia may not have been prevalent in the studied population at the time of assessment. In terms of hematopoietic cell transplantation (HCT), only one adult patient was reported receiving this treatment (X^2^ = 1.06, *p* = 0.79), indicating that this potentially curative option remains underutilized across all age groups. Barriers to transplantation may include the availability of suitable donors, the complexity of the procedure, and the need for extensive pre-transplant evaluations [81,82]. Moreover, HCT is a critical and potentially curative treatment for severe SCD (e.g., stroke, recurrent pain, or chronic transfusion), offering hope for the long-term amelioration of disease manifestations [81,82]. HCT from HLA-matched sibling donors has shown excellent outcomes, with an event-free survival rate of 82% [83]. Gene therapy approaches are also emerging as promising alternatives [84]. Despite these developments, HCT for SCD presents ethical challenges and complex decision-making processes [85]. Improved patient and caregiver education about HCT is needed, as many are unaware of its curative potential [86]. In contrast, blood transfusions were reported in a significant proportion of patients, with 71.4% of children, 61.1% of adolescents, 44.4% of adults, and 76.9% of middle-aged patients receiving this treatment, highlighting a clear difference across age groups (X^2^ = 11.02, *p* = 0.012). The high prevalence of blood transfusions, especially in children and middle-aged patients, underscores their importance in managing severe anemia and preventing complications such as ischemic stroke in these vulnerable groups [87,88].

Several limitations must be acknowledged in this study. Firstly, the retrospective design may introduce bias related to data collection and patient recall. In addition, the study’s reliance on existing medical records potentially limits the depth of clinical information available, particularly regarding patient-reported outcomes. The geographic focus on Taif City is a further perceived limitation as this geographical location restricts the generalizability of the findings to other regions or populations whose demographics may differ. This study did not specifically analyze gender differences in the symptoms and complications of SCD, which represents a limitation that future research should address. Furthermore, HCT is currently the only curative treatment for SCD; however, only one patient in our study received this intervention. Future research should investigate the challenges associated with the utilization of HCT for SCD in greater depth to develop effective strategies for improving its accessibility and implementation. Lastly, the relatively small sample size, particularly in certain age groups, is a considerable limitation of the current study, as it lowers the statistical power to detect subtle differences in outcomes and complications. Accordingly, in an effort to eliminate some of these limitations, future prospective studies incorporating a larger and more diverse population would be beneficial in addressing these limitations and further elucidating the complexities of SCD.

## 5. Conclusions

**Figure 3:**
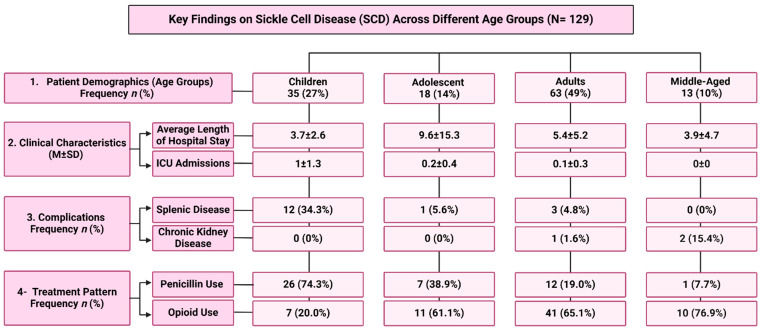
The flowchart shows the main findings of the study.

The study reveals significant differences in sickle cell disease (SCD) across various age groups, underscoring the necessity for age-specific approaches in clinical management. Our findings illustrate the notable heterogeneity in disease characteristics and clinical outcomes among children, adolescents, adults, and middle-aged individuals with SCD. Distinct patterns emerged concerning hospital stays, ICU admissions, complications, and treatment modalities, emphasizing the importance of tailored management for this diverse patient population (see Figure 3). A key takeaway is the heightened disease severity and healthcare utilization observed in pediatric patients, who demonstrated the highest rates of ICU admissions, highlighting the need for specialized care and vigilant monitoring to address their unique challenges. Additionally, our study identifies differences in complication profiles by age, with splenic disease prevalent in children and chronic kidney disease primarily affecting adults and middle-aged individuals. This information is crucial for guiding targeted screening and management strategies to mitigate potentially life-threatening complications. Furthermore, our analysis of treatment patterns reveals significant variations in the use of therapies such as penicillin and opioids. The increased reliance on penicillin in children and opioid use in middle-aged individuals points to the need for pharmacological management tailored to age-specific risks and benefits. These findings reinforce the importance of patient-centered, age-appropriate care for individuals with SCD. By acknowledging the diverse clinical manifestations and healthcare needs across age groups, healthcare providers can optimize disease management, improve patient outcomes, and enhance overall quality of life. Future research should delve deeper into the mechanisms underlying age-related differences in SCD, aiming to develop innovative, personalized therapeutic strategies. Moreover, longitudinal studies that track disease progression and long-term outcomes will provide valuable insights to refine comprehensive care models for this patient population.

## Figures and Tables

**Table 1:** Description of demographic data in all studied patients.

Age Group	Male (N = 60)	Female (N = 69)	Frequency *n* (%)
Children (0–12) years	16	19	35 (27%)
Adolescents (13–19) years	9	9	18 (14%)
Adults (20–39) years	31	32	63 (49%)
Middle-Aged (40–55) years	4	9	13 (10%)
Frequency *n* (%)	60 (46.5%)	69 (53.5%)	129 (100%)
Age (Mean ± SD)	23 ± 11.7	23 ± 14.2	23 ± 13

**Table 2:** Comparison of gender among all patient age groups.

		Children Frequency *n* (%)	Adolescent Frequency *n* (%)	Adults Frequency *n* (%)	Middle-Aged Frequency *n* (%)	X^2^	*p*-Value
Gender	Male	16 (45.7)	9 (50)	31 (49.2)	4 (30.8)	1.6	0.7
	Female	19 (54.3)	9 (50)	32 (50.8)	9 (69.2)		

X^2^: Chi-square test.

**Table 3:** Comparison of clinical characteristics of all patient age groups.

Hospitalization and Patient Outcome Parameters (Mean ± SD)	Children (N = 35)	Adolescents (N = 18)	Adults (N = 63)	Middle-Aged (N = 13)	F	*p*-Value
Length of Hospital Stay	3.7 ± 2.6	9.6 ± 15.3	5.4 ± 5.2	3.9 ± 4.7	2.982	0.034
Number of Hospital Admissions	2.4 ± 1.3	1.7 ± 1	2.1 ± 2.8	1.4 ± 0.7	0.889	0.45
Number of ICU Admissions	1 ± 1.3	0.2 ± 0.4	0.1 ± 0.3	0 ± 0	4.727	0.004
O_2_ Saturation	94 ± 5.5	94 ± 3.1	95 ± 3.7	95 ± 3.6	0.31	0.82

F: value of ANOVA test. ICU: Intensive care unit. SD: Standard deviation.

**Table 4:** Comparison of laboratory data for all age groups of patients.

Lab Results (Mean ± SD)	Children (N = 35)	Adolescents (N = 18)	Adults (N = 63)	Middle-Aged (N = 13)	F	*p*-Value
WBCs	11.6 ± 6.9	13.0 ± 4.6	14.6 ± 6.8	14.9 ± 3.6	1.76	0.16
Reticulocyte count	0.4 ± 0.2	0.8 ± 0	0.3 ± 0.2	0.2 ± 0	1.214	0.32
RBCs	3.1 ± 0.9	5 ± 3.7	3.6 ± 1.3	4.7 ± 3.9	2.449	0.072
Platelet	332 ± 171	426 ± 223	414 ± 199	489 ± 263	2.210	0.09
Hematocrit	0.25 ± 5.71	0.3 ± 4.56	0.27 ± 8.54	0.21 ± 3.68	0.0004	0.99
Hemoglobin	10.22 ± 14.67	7.35 ± 3.22	10.42 ± 11.21	8.16 ± 2.64	0.467	0.705
Creatinine	0.3 ± 0.1	1.8 ± 3.2	0.9 ± 1.3	2.2 ± 2.2	4.416	0.006
ALT	21.5 ± 18.2	19.6 ± 5.7	26.6 ± 20.6	34.8 ± 31	1.538	0.21
AST	46.1 ± 24	78.6 ± 66.3	37.4 ± 28.5	35 ± 24.1	5.606	0.001
Total bilirubin	44.6 ± 33.5	37.1 ± 55.4	79.6 ± 188.6	69.8 ± 128.5	0.554	0.65
Direct bilirubin	10.2 ± 7.8	8.8 ± 16.2	37.6 ± 129.5	19.3 ± 40.8	0.693	0.56
LDH	715 ± 374	1226 ± 738	580 ± 375	735 ± 391	4.950	0.004

F: value of ANOVA test. LDH: Lactate dehydrogenase.

**Table 5:** Comparison of complications of all age groups of patients.

Complications	Children (N = 35) Frequency *n* (%)	Adolescents (N = 18) Frequency *n* (%)	Adults (N = 63) Frequency *n* (%)	Middle-Aged (N = 13) Frequency *n* (%)	X^2^	*p*-Value
Acute Chest Syndrome	7 (20)	1 (5.6)	7 (11.1)	1 (7.7)	3	0.39
Avascular Osteonecrosis	0 (0)	0 (0)	5 (7.9)	0 (0)	5.4	0.14
Acute Kidney Injury	0 (0)	0 (0)	1 (1.6)	1 (7.7)	4.05	0.26
Chronic Kidney Disease	0 (0)	0 (0)	1 (1.6)	2 (15.4)	11.2	0.01
Pulmonary HTN	0 (0)	0 (0)	1 (1.6)	1 (7.7)	4.05	0.26
Delayed Growth and Development	1 (2.9)	0 (0)	1 (1.6)	0 (0)	0.88	0.8
Pain	24 (68.6)	10 (55.6)	42 (66.7)	8 (61.5)	1.05	0.79
Ischemic Stroke	0 (0)	0 (0)	1 (1.6)	0 (0)	1.06	0.79
Osteomyelitis	2 (5.7)	0 (0)	1 (1.6)	1 (7.7)	2.8	0.43
Vascular Occlusive Crisis	15 (42.9)	6 (33.3)	28 (44.4)	4 (30.8)	1.4	0.72
UTIs	1 (2.9)	0 (0)	0 (0)	0 (0)	2.7	0.44
Gallstone	4 (11.4)	1 (5.6)	5 (7.9)	0 (0)	1.9	0.6
Splenic Disease	12 (34.3)	1 (5.6)	3 (4.8)	0 (0)	21.4	<0.001
Priapism	1 (2.9)	0 (0)	1 (1.6)	0 (0)	0.88	0.8

X^2^: Chi-square test. HTN: Hypertension. UTIs: Urinary tract infections.

**Table 6:** Comparison of medications used in patients of different age groups.

Pharmacological Treatment		Children (N = 35) Frequency *n* (%)	Adolescents (N = 18) Frequency *n* (%)	Adults (N = 63) Frequency *n* (%)	Middle-Aged (N = 13) Frequency *n* (%)	X^2^	*p*-Value
Folic Acid	Yes	31 (88.6)	16 (88.9)	49 (77.8)	11 (84.6)	2.45	0.49
	No	4 (11.4)	2 (11.1)	14 (22.2)	2 (15.4)		
Hydroxyurea	Yes	22 (62.9)	11 (61.1)	32 (50.8)	7 (53.8)	1.57	0.67
	No	13 (37.1)	7 (38.9)	31 (49.2)	6 (46.2)		
ACE Inhibitors	Yes	0 (0.0)	0 (0.0)	1 (1.6)	2 (15.4)	11.2	0.011
	No	35 (100)	18 (100)	62 (92.4)	11 (84.6)		
EPO	Yes	1 (2.9)	0 (0.0)	0 (0.0)	0 (0.0)	2.7	0.44
	No	34 (97.1)	18 (100)	63 (100)	13 (100)		
Penicillin	Yes	26 (74.3)	7 (38.9)	12 (19.0)	1 (7.7)	34.9	<0.001
	No	9 (25.7)	11 (61.1)	51 (81.0)	12 (92.3)		
Iron Chelation	Yes	4 (11.4)	0 (0.0)	0 (0.0)	0 (0.0)	11.1	0.011
	No	31 (88.6)	18 (100)	63 (100)	13 (100)		
Opioids	Yes	7 (20.0)	11 (61.1)	41 (65.1)	10 (76.9)	22.5	<0.001
	No	28 (80.0)	7 (38.9)	22 (34.9)	3 (23.1)		

X^2^: Chi-square test.

**Table 7:** Comparison of non-pharmacological treatment among all patients age groups.

Non-Pharmacological Treatment		Children (N = 35) Frequency *n* (%)	Adolescents (N = 18) Frequency *n* (%)	Adults (N = 63) Frequency *n* (%)	Middle-Aged (N = 13) Frequency *n* (%)	X^2^	*p*-Value
O_2_ Therapy	Yes	5 (14.3)	2 (11.1)	4 (6.3)	0 (0.0)	3.2	0.36
	No	30 (85.7)	16 (88.9)	59 (93.7)	13 (100)		
Hematopoietic Cell Transplant (HCT)	Yes	0 (0.0)	0 (0.0)	1 (1.6)	0 (0.0)	1.06	0.79
	No	35 (100)	18 (100)	62 (92.4)	13 (100)		
Blood Transfusion	Yes	25 (71.4)	11 (61.1)	28 (44.4)	10 (76.9)	11.02	0.012
	No	10 (28.6)	7 (38.9)	35 (55.6)	3 (23.1)		

X^2^: Chi-square test.

## Data Availability

The data that support the findings of this study are available from the corresponding author, Dr. Daniyah A. Almarghalani, upon reasonable request.

## Supplementary Materials

The following supporting information can be downloaded at: https://www.mdpi.com/article/10.3390/jcm13237224/s1, Figure S1: Complications associated with SCD, including pain, vascular occlusive crisis, acute chest syndrome, splenic diseases, gallstones, and jaundice; Table S1: Description of complications in all studied patients; Table S2: Comparison of co-morbidities and mortality of all age groups of patients; Table S3: Comparison of all age groups of patients related to vaccine type.
