# Supplementary material for: Clinical Insights into Sickle Cell Disease: A Comprehensive Multicenter Retrospective Analysis of Clinical Characteristics and Outcomes Across Different Age Groups
Source: J Clin Med. 2024 Nov 28;13(23):7224. doi: 10.3390/jcm13237224 (PMC11641892; doi:10.3390/jcm13237224)
Supplement: Supplementary file 1 [file jcm-13-07224-s001.zip › jcm-3335725-supplementary.pdf]

Supplementary Table S1. Description of complications in all studied patients.

Complications	All Patients (N= 129) Frequency *n* (%)	Complications	All Patients (N= 129) Frequency *n* (%)
**Pain**	84 (65.1)	**Acute Kidney Disease**	2 (1.6)
**Vascular Occlusive Crisis**	53 (41.1)	**Pulmonary Hypertension**	2 (1.6)
**Acute Chest Syndrome**	16 (12.4)	**Delayed Growth and Development**	2 (1.6)
**Splenic Disease**	16 (12.4)	**Acute Cholecystitis**	2 (1.6)
**Gallstone**	10 (7.8)	**Priapism**	2 (1.6)
**Jaundice**	7 (5.4)	**Osteoporosis**	1 (0.8)
**Avascular Osteonecrosis**	5 (3.9)	**Shortness of Breath**	1 (0.8)
**Osteomyelitis**	4 (3.1)	**Epilepsy**	1 (0.8)
**Hemolytic Crisis**	3 (2.3)	**UTIs**	1 (0.8)
**Chronic Kidney Disease**	3 (2.3)	**Ischemic Stroke**	1 (0.8)

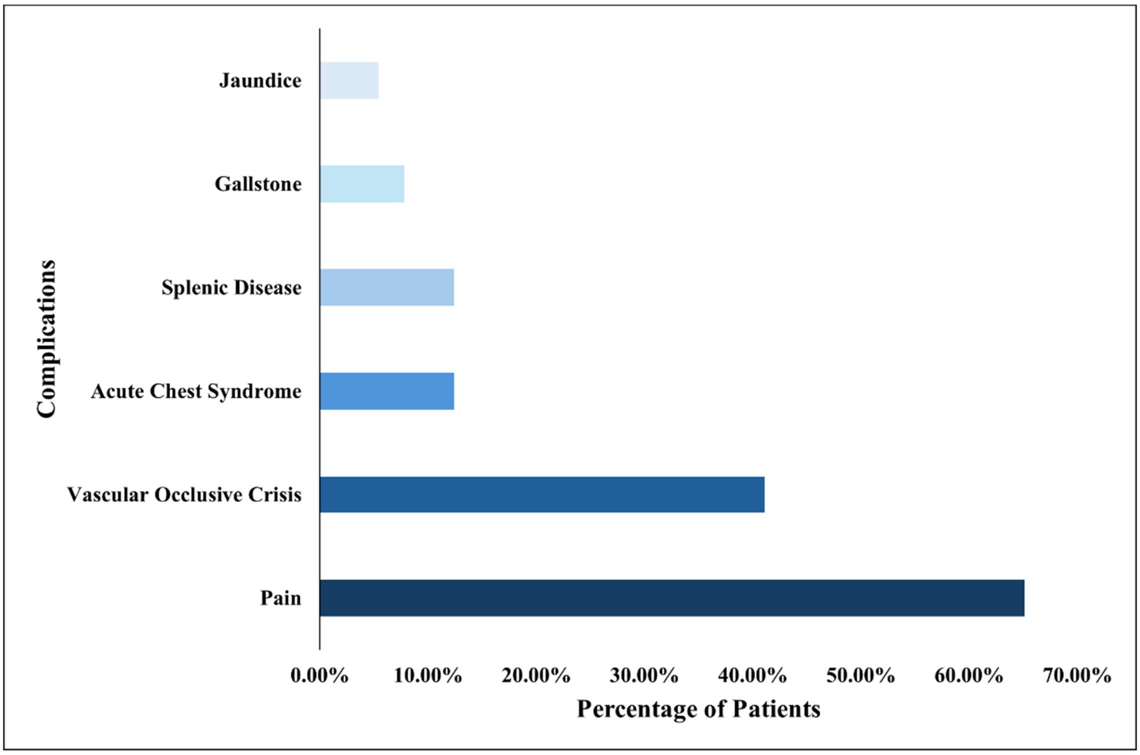

Supplementary Figure S1. Complications associated with SCD, including pain, vascular occlusive crisis, acute chest syndrome, splenic diseases, gallstones, and jaundice.

Supplementary Table S2. Comparison of co-morbidities and mortality of all age groups of patients.

Co-morbidities	Children (N= 35) Frequency *n* (%)	Adolescents (N = 18) Frequency *n* (%)	Adults (N= 63) Frequency *n* (%)	Middle-Aged (N = 13) Frequency *n* (%)	X^2	*p*-value
**Diabetes**	1 (2.9)	0 (0)	3 (4.8)	1 (7.7)	1.5	0.7
**Hypertension**	0 (0)	0 (0)	3 (4.8)	2 (15.4)	6.9	0.08
**Mortality (Death)**	1 (2.9)	1 (5.6)	0 (0)	0 (0)	3.5	0.32

Abbreviation: X^2: Chi-square test.

3.8. Vaccine Analysis in Patients with SCD

The analysis of vaccination status revealed significant differences among age groups. Only 20.0% of children and 16.7% of adolescents received the pneumococcal vaccine, with no adults or middle-aged patients vaccinated (X^2 = 15.7, P = 0.001). Similarly, the meningococcal vaccine was administered to 17.1% of children and 16.7% of adolescents, while none of the adults or middle-aged patients received this vaccine (X^2 = 13.9, P = 0.003). The H. Influenza Type B vaccine was given to 14.3% of children and 5.6% of adolescents, with no vaccinations recorded in the adult or middle-aged groups (X^2 = 11.1, P = 0.011) (Supplementary Table S3).

Supplementary Table S3: Comparison of all age groups of patients related to vaccine type.

Vaccine Type		Children (N= 35) Frequency *n* (%)	Adolescents (N= 18) Frequency *n* (%)	Adults (N= 63) Frequency *n* (%)	Middle-Aged (N= 13) Frequency *n* (%)	X^2	*p*-value
**Pneumococcal Vaccine**	**Yes**	7 (20.0)	3 (16.7)	0 (0.0)	0 (0.0)	15.7	**0.001**
	**No**	28 (80.0)	15 (83.3)	63 (100)	13 (100)		
**Meningococcal Vaccine**	**Yes**	6 (17.1)	3 (16.7)	0 (0.0)	0 (0.0)	13.9	**0.003**
	**No**	29 (82.9)	15 (83.3)	63 (100)	13 (100)		
**H. Influenza Type B Vaccine**	**Yes**	5 (14.3)	1 (5.6)	0 (0.0)	0 (0.0)	11.1	**0.011**
	**No**	30 (85.7)	17 (94.4)	63 (100)	13 (100)		

Abbreviation: X^2: Chi-square test.
